# *In vitro* activity of cefiderocol against *Pseudomonas aeruginosa* isolated from cystic fibrosis patients

**DOI:** 10.1128/spectrum.03047-23

**Published:** 2023-11-20

**Authors:** Marguerite L. Monogue, Dhara Desai, Christine A. Pybus, James M. Sanders, Andrew E. Clark, David E. Greenberg

**Affiliations:** 1 Department of Pharmacy, University of Texas Southwestern Medical Center, Dallas, Texas, USA; 2 Department of Internal Medicine, Infectious Diseases, and Geographic Medicine, University of Texas Southwestern Medical Center, Dallas, Texas, USA; 3 Department of Microbiology, University of Texas Southwestern Medical Center, Dallas, Texas, USA; 4 Department of Pathology, University of Texas Southwestern Medical Center, Dallas, Texas, USA; University of Texas Southwestern Medical Center, Dallas, Texas, USA

**Keywords:** cefiderocol, cystic fibrosis, *Pseudomonas aeruginosa*, *in vitro*

## LETTER

*Pseudomonas aeruginosa* (PSA) is a common cause of acute pulmonary exacerbations in adult cystic fibrosis (CF) patients. In this setting, culture isolates are often multidrug resistant (MDR) or exhibit difficult-to-treat resistance (DTR) due to frequent antimicrobial exposure, resulting in the use of last-line or novel therapies (1). Cefiderocol (CFDC) appears less susceptible to acquired resistance mechanisms such as efflux pump upregulation, porin loss, and AmpC hyperexpression mediating imipenem-relebactam (I-R), ceftolozane-tazobactam (C-T), and ceftazidime-avibactam (CZA) resistance (2 – 7). The purpose of this study was to compare the *in vitro* potency of CFDC with other broad-spectrum anti-pseudomonal agents (I-R, C-T, CZA) using a collection of clinical PSA isolates recovered from adult CF respiratory specimens.

A total of 31 adult CF PSA isolates were selected from an internal repository (8). These respiratory isolates were collected from inpatient clinical samples from 2019 to 2021. Isolates were selected for testing in chronological order. Minimum inhibitory concentrations (MICs) to CFDC were derived in triplicate via broth microdilution, with the modal MIC reported (9). Susceptibility testing was repeated if no mode was available. If modal distribution could not be established following repeat testing, the median MIC was selected. Associated CLSI, EUCAST, and FDA interpretive breakpoints were applied (9 – 11). MIC panels were prepared in-house. Iron-depleted cation-adjusted Mueller-Hinton broth (ID-CAMHB, Thermofisher scientific) was used to determine CFDC MICs. Individual imipenem, relebactam, ceftazidime, and avibactam powders were obtained from MedChem Express (Lot# MCE 91445, MCE 114223, MCE 118531, MCE 22312, respectively). Ceftolozane and tazobactam powders were obtained from Merck (Lot# 44042000041, 5/08, respectively), and CFDC was obtained from Shionogi (Lot# 0019, exp 8/25).

Overall, CFDC retained the greatest *in vitro* potency compared with I-R, C-T, and CZA (Table 1). The MIC_50_/MIC_90_ of CFDC was 0.25/16 µg/mL, respectively. The MIC_50_/MIC_90_ of I-R, C-T, and CZA was 2/32, 1/128, and 2/128 µg/mL, respectively (Table 2). Of note, a quarter of tested PSA isolates were non-susceptible to meropenem (MIC > 2 µg/mL, CLSI and EUCAST breakpoint). Against meropenem non-susceptible isolates, CFDC retained the lowest MIC_50_ compared with the other novel agents (CFDC MIC_50_/MIC_90_ of 1/128 µg/mL; 75% susceptible per CLSI; Table 3).

**TABLE 1:** MICs (µg/mL) of CFDC and comparators (I-R, C-T, CZA, and MEM) against a collection of 31 PSA isolates

Isolate	CFDC MIC	I-R MIC	C-T MIC	CZA MIC	MEM MIC
518	4	4	≥128	≥128	0.5
1383	32	32	4	4	2
159	1	64	16	64	4
1306	4	16	16	16	8
2469	2	2	4	2	0.25
2565	1	16	≥128	≥128	16
2682	≤0.03	32	1	2	2
2791	0.125	2	2	4	0.125
3358	≥128	32	≥128	64	8
3381	≤0.03	4	0.5	1	0.125
4216	0.5	32	1	4	4
4690	≥128	8	≥128	≥128	4
5119	16	16	64	32	2
4717	0.5	4	1	2	0.0625
875	2	2	2	2	2
2782	≤0.03	≤0.03	1	0.25	<0.0625
774	2	0.5	0.5	2	0.0625
955	≤0.03	0.125	0.25	1	1
5529	≤0.03	0.25	0.25	1	<0.0625
6653	1	32	64	32	8
5431	0.125	0.25	0.5	2	0.25
5299	≤0.03	0.125	0.25	1	<0.0625
5275	0.125	2	1	2	2
5234	≤0.03	0.125	0.25	0.25	0.5
5559	0.25	2	0.5	1	0.125
5549	2	0.25	1	2	<0.0625
6831	≤0.03	0.25	0.25	≤0.03	<0.0625
5455	≤0.03	0.25	0.5	1	0.25
5441	≤0.03	0.5	0.5	2	0.0625
5318	≤0.03	4	1	2	0.125
6186	0.125	16	64	≥128	8

^*a*^ MEM, meropenem.

**TABLE 2:** The MIC_50_ and MIC_90_ (µg/mL) of CFDC and later generation beta-lactam/beta-lactamase inhibitor combinations against CF PSA isolates (*n* = 31)^*a*^

	CFDC	I-R	C-T	CZA	MEM
MIC_50_	0.25	2	1	2	0.5
MIC_90_	16	32	≥128	≥128	8

^*a*^ MEM, meropenem.

**TABLE 3:** Percent susceptibility of novel antimicrobials against meropenem non-susceptible CF PSA isolates (*n* = 8)^*a*^

	CFDC	I-R	C-T	CZA
CLSI	75	0	13	13
EUCAST	63	0	13	13
FDA	68	–^*b*^	–	–

^*a*^ CLSI susceptible breakpoint (µg/mL): CFDC ≤ 4, I-R ≤ 2/4, C-T ≤ 4/4, and CZA ≤ 8/4. EUCAST susceptible breakpoint (µg/mL): CFDC ≤ 2 , I-R ≤ 2/4, C-T ≤ 4/4, and CZA ≤ 8/4. FDA susceptible breakpoint (µg/mL): CFDC ≤ 1, I-R, C-T, CZA CLSI M100 is recognized.

^*b*^ –, not available.

Antibiotic management of pulmonary exacerbations in the setting of CF is challenging as the etiologic agents often exhibit MDR. CFDC has enhanced activity against MDR PSA and is, therefore, an attractive option for intervention; albeit, CFDC resistance may occur (12, 13). The observed amount of CFDC resistance in our adult CF isolates reflects that of pediatric CF isolates (14). Current guidance from the IDSA recommends C-T, CZA, or I-R for systemic infections caused by DTR-PSA, whereas CFDC is mentioned as an alternative option (13). Routine susceptibility testing for these novel agents in the setting of MDR pathogens will help guide and expedite antimicrobial therapy. Following C-T exposures, CFDC maintains the greatest *in vitro* potency against PSA compared with C-T, CZA, and I-R. However, changes in target binding sites of PSA-derived AmpC β-lactamases may result in an increase in CFDC MICs (2). Furthermore, following CFDC exposure, alterations in bacterial outer membrane proteins enabling uptake of specific siderophore–iron complexes across the bacterial membrane may occur leading to CFDC resistance (15). Our study is limited by the small sample size and single institutional cohort. Additionally, we did not quantify antimicrobial exposure to correlate with observed resistance. Future work is aimed at characterizing the molecular mechanisms underpinning resistance to these novel agents in this collection of PSA isolates recovered from the CF lung.
